# Fueling Soccer Players: A Scoping Review and Audit of Literature Related to Soccer-Specific Guidelines for Carbohydrate Intake

**DOI:** 10.1007/s40279-025-02224-3

**Published:** 2025-04-22

**Authors:** Wee Lun Foo, Emma Tester, Graeme L. Close, José L. Areta, James P. Morton

**Affiliations:** 1 Research Institute for Sport and Exercise Sciences (RISES), Liverpool John Moores University, Byrom Street, Liverpool, L3 3AF UK; 2 Tottenham Hotspur Football Club, Enfield, London, EN2 9AP UK

## Abstract

**Background:**

Professional soccer players are advised to consume 3–8 g kg^–1^ body mass day^–1^ of carbohydrate (CHO) on the basis of training demands, fixture schedule and personal objectives. However, owing to the lack of randomized controlled trials on elite players, these guidelines largely rely on data interpretation and practitioner experience.

**Objective:**

To identify the gaps in existing literature that inform CHO guidelines for soccer players.

**Methods:**

A scoping review was conducted without date restrictions up to 21 March 2024, employing a three-step search strategy to identify relevant English-language primary and secondary articles through PubMed and reference searching. Data were extracted using a standardized audit tool from studies assessing direct and indirect impacts of CHO on soccer players’ performance and health.

**Results:**

Within 258 studies identified, experimental studies were the most common (~ 36%), followed by observational (~ 33%) and narrative reviews (~ 26%), with systematic reviews, meta-analyses and case studies making up the rest (~ 5%). Most observational studies were field-based (~ 98%), while experimental studies were laboratory-based (~ 75%). Among 4475 participants, ~ 16% were female, and only ~ 12% of the original research was exclusively conducted on female players. Observational studies included developmental (~ 52%) and professional players (~ 31%), whereas experimental studies primarily featured recreationally active (~ 40%) and collegiate/university participants (~ 26%). Key research topics were ‘dietary intake’ (~ 52%) and “energy expenditure and dietary intake” (~ 30%) for observational studies and ‘CHO interventions’ (~ 74%) for experimental studies. Only eight experimental studies exclusively involved professional players, focusing on CHO intervention (*n* = 7) and CHO co-ingestion (*n* = 1). Narrative reviews were published in journals with higher impact factor (4.1 ± 6.4) than were observational studies (3.2 ± 1.6, *p* < 0.001) and experimental studies (3.4 ± 1.6, *p* < 0.001). Narrative reviews had the most studies, with Altmetric scores ≥ 20 (*n* = 26), followed by experimental (*n* = 16) and observational studies (*n* = 14).

**Conclusions:**

Current CHO guidelines for elite soccer players lack experimental research specific to professional and world-class players. More field-based experimental trials involving elite soccer players are required to ensure evidence-based CHO recommendations.

## Key Points

**Table:** 

This scoping review and audit of carbohydrate research for soccer shows a predominance of laboratory-based experimental studies and field-based observational studies, while the scarcity of field-based experimental trials involving professional and world-class soccer players highlights the need for such research to enhance the translational applicability of carbohydrate guidelines for this population.
Narrative reviews occupied a significant space in the carbohydrate literature for soccer and tended to be published in higher impact factor journals and receive more Altmetric attention compared with original research; therefore, this trend may incentivize researchers to publish reviews over original studies, potentially slowing progress in advancing practical, evidence-based carbohydrate guidelines in soccer.
Female soccer players are notably underrepresented in carbohydrate research, resulting in current Union of European Football Associations (UEFA) nutrition guidelines for females being largely extrapolated from male-focused data; this emphasizes the urgent need for targeted, evidence-based studies to inform sex-specific nutritional guidelines for female soccer players.

## Introduction

In 1973, the late Professor Bengt Saltin published the first data evaluating the effects of muscle glycogen availability on soccer-specific performance [1]. These data demonstrated that players (*n* = 5) who commenced the match with low muscle glycogen covered less total distance (9700 m versus 12,000 m), particularly in the second half (4100 m versus 5900 m) and spent more time walking (50% versus 27%) and less time sprinting (15% versus 24%) than players with high muscle glycogen (*n* = 4). Such seminal data paved the way for the development of soccer-specific sport nutrition guidelines, and in the 50 years since, the research base with application to intermittent exercise and soccer has grown considerably.

In one of the earliest nutritional guidelines published in 1994, soccer players were advised to maintain a diet comprising 55–65% carbohydrate (CHO) on training days, with a specific recommendation to consume between 7 and 10 g kg^−1^ body mass (BM) day^−1^ to optimize glycogen stores ahead of a game [2]. In 2006, players received more nuanced guidance, suggesting a recommended range of 5–7 g kg^−1^ BM day^−1^ CHO on moderate training days and up to 12 g kg^−1^ BM day^−1^ CHO on intense training days or when preparing for a match [3]. The most recent nutritional guidelines for soccer players were subsequently published in 2020, in an expert led group statement (comprising 31 authors with both research and applied practitioner experience) that was endorsed by the Union of European Football Associations (UEFA) [4]. In this latest statement, a recommended range of daily CHO intake of 3–8 g kg^−1^ BM day^−1^ was suggested, emphasizing that daily CHO intake should also be adjusted day-by-day in accordance with the training demands, fixture schedule and any player specific objectives.

Despite the publication of such recommendations, the senior author (J.P.M.) of the present paper (also a co-author on the UEFA group statement) can recall the sparsity of the evidence base to underpin such recommendations, especially in relation to randomized controlled trials conducted on elite players. Rather, the publication of such recommendations was not only based on a combination of interpretation of available data but also on practitioner experience when working with elite players. Such a critique of literature is not unique to soccer and, indeed, has also been recognized in the wider context of ‘sport nutrition’ guidelines in general [5]. Indeed, there has been a notable shift in research focus over the last 5 years, with greater emphasis on reviews rather than original research in sports nutrition [5]. For example, ~ 20% of published articles have been reviews, ~ 6% being meta-analyses. Although reviews provide valuable synthesis, they carry the risk of biased interpretation and potential distortion of original information through repeated paraphrasing [6]. Thus, the significance of continuing original research in advancing the field cannot be overstated. Consequently, much of the practical application of sport-specific nutritional guidelines (especially in the context of soccer) is left to the individual interpretation of those practitioners operating in the field. For instance, the current CHO guidelines for soccer players, recommending 3–8 g kg^−1^ BM day^−1^, encompass a wide range that includes both low and high CHO intakes. This approach essentially leaves it to the discretion of the nutritionist to determine the appropriate amount for each player. Furthermore, assessments of dietary intakes of players (albeit self-reported) continue to highlight that players do not readily meet recommended CHO intakes, especially in preparation for match play [7], likely owing to a combination of complex factors, including athlete and stakeholder education, beliefs and the wider nutrition culture of the specific environment [8–11].

In an attempt to better inform evidence-based soccer-specific nutrition guidelines, the aim of the present study was to conduct a scoping review and research audit of literature (both reviews and original research) with relevance to soccer-specific guidelines for CHO intake. To this end, we used a well-established scoping review framework [12], combined with a recently published research audit methodology [13], to perform an extensive audit of literature pertaining to adult male, female and junior soccer players. Importantly, this review identifies gaps in literature and presents those research areas with the greatest scope to inform practice. In this way, it is hoped that the present paper stimulates concerted and collaborative research worldwide to ensure that future soccer-specific nutrition guidelines are supported by a stronger research base.

## Methods

This scoping review followed the well-established five stages framework as suggested by Arksey and O’Malley [12], integrating the audit protocol from Smith et al. [13]. Information specific to the current review is detailed below.

### Stage 1: Identification of the Research Question

Considering the context, a broad research question was decided upon:

‘What are the key gaps in the existing body of research that inform carbohydrate guidelines for soccer players, considering different study designs, competitive levels, research themes and demographic groups?’

### Stage 2: Identification of Relevant Studies

The following inclusion and exclusion criteria were established through discussion between the author group.

#### Inclusion Criteria

- All age groups and both female and male participants.
- Research articles are not limited by geographical location or setting.
- Published in the English language.
- Full text links are available.
- Any levels of soccer (e.g. sedentary, recreationally active, collegiate/university, developmental, semi-professional, professional and world-class).
- Direct measurements of performance or health parameters, and indirect contributions/markers of performance/health.
- Research published without date restrictions and current to 21 March 2024.
- Sources of information—including primary and secondary research studies, reviews, systematic reviews, scoping reviews, case studies, meta-analyses and guidelines.

#### Exclusion Criteria

-  ≥ 50 years of age.
- Presence of lifestyle diseases (e.g. obesity, hypertension, diabetes) or smoking.
- Failure to investigate CHO or CHO-related markers as the primary outcome/independent variable.
- Outcomes irrelevant to areas of interest.
- Grey literature (i.e. unpublished and ongoing trials, annual reports, dissertations and conference proceedings).

### Search Strategies and Database

The search strategy aimed to discover published studies. An electronic literature search of PubMed was conducted using the terms: ‘(Carbohydrate OR Glycogen OR Glucose OR Sugar) AND (Soccer OR Football OR Team Sports OR Intermittent Running) NOT (Diabetes)’. Following the initial search, the reference lists of all included articles were screened for further relevant papers that were not detected in the primary search.

### Stage 3: Study Selection

**Fig. 1:**
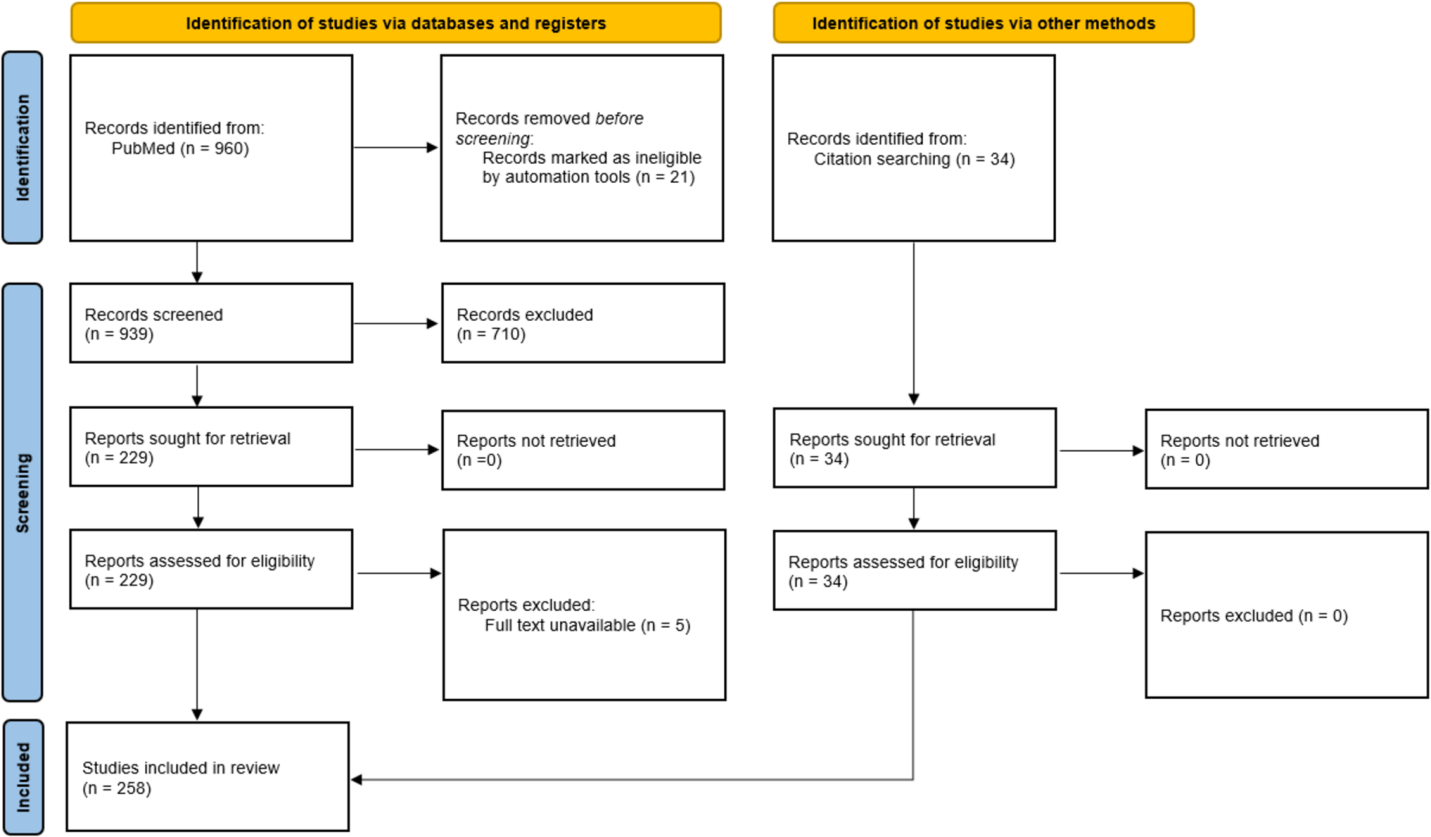
PRISMA-ScR flowchart illustrating the different phases of the search and study selection

Following the search, all identified citations were collated and duplicates removed. The lead reviewer (W.L.F.) then examined the titles, abstracts and reference lists of each study to identify the relevant studies. Potentially relevant sources were retrieved in full. Full studies were subsequently screened for their relevance to the selection criteria. The secondary reviewer (J.P.M.) completed the same process on a random sample of 10% of the titles with concordance > 97%. Where a decision was not reached at any stage of the selection process, it was resolved through discussion. The results of this search and study inclusion process are reported in full in the final scoping review and are presented in a Preferred Reporting Items for Systematic Reviews and Meta-Analyses extension for Scoping Review (PRISMA-ScR) flow diagram (Fig. 1) [14].

### Stage 4: Charting the Data

Charting tables to record and collect extracted data from included studies were developed. The lead reviewer (W.L.F.) conducted the data extraction process, systematically collecting information to a predefined template. The secondary reviewer (J.P.M.) cross-verified 10% of the lead reviewer’s data extraction to ensure accuracy and reliability. Any discrepancies were resolved through discussion. This structured approach to data extraction was facilitated through a comprehensive datasheet that encompassed all details and essential information. With the use of the protocol outlined in Smith et al. [13], details of the following metrics were extracted: (a) research types: review (narrative), review (systematic), review (meta-analysis), observational, experimental/clinical trials and case studies; (b) research settings: field or laboratory-based; (c) participants’ demographics: sex (male or female), age groups (adults—aged ≥ 18 years old or adolescents—aged 10–17 years old); (d) athletic caliber (Table 1): sedentary, recreationally active, collegiate/university, developmental, semi-professional, professional and world-class; (e) research topics; (f) journal publication dates and study impact (Altmetric scores and journal impact factor (IF)); and (g) mean sample size. In the present review, it was not appropriate to exclude sedentary participants owing to the relevance of mechanistic studies not requiring an exercise condition. Studies involving separate investigations were included in the audit, and their metrics counted separately.

**Table 1:** Participant classification framework in soccer

Competitive level	Descriptions
World-class	World-class players include the most competitive soccer players competing in the most exclusive soccer leagues. World-class players are usually “starters” for the top teams in the top five leagues in the world and/or the “starters” for the top ten ranked national soccer teams in the world Men: English Premier League, Serie A, La Liga, Bundesliga and Ligue 1 Women: Women’s Super League, Liga F, Division 1 Féminine, Frauen-Bundesliga and National Women’s Soccer League
Professional	Professional players include players competing in a professional league with a full-time contract
Semi-professional	Semi-professional players include players who are not full-time but still receive regular payments from the club
Collegiate/university	Players compete in university soccer teams; varsity teams and National Collegiate Athletics Association (NCAA)
Developmental	Participants in the academy or “second team” of professional soccer clubs
Recreationally active	Meet World Health Organization minimum activity guidelines: adults aged 18–64 years old completing at least 150–300 min moderate-intensity activity or 75–150 min of vigorous-intensity activity a week, plus muscle-strengthening activities 2 or more days a week [47]. May participate in multiple sports/forms of activity
Sedentary	Do not meet minimum activity guidelines. Occasional and/or incidental physical activity (e.g. walking to work, household activities)

### Stage 5: Collating, Summarizing and Reporting the Results

Methods undertaken in the protocol by Smith et al. [13] permitted us to collate and audit existing knowledge on this body of literature in four different domains:

1. Study types and research settings
2. Population and sample size
3. Athletic caliber and research topics
4. Journal and study impact

In this scoping review, we aimed to (1) identify gaps in existing literature related to CHO and soccer to guide future research directions and (2) report and summarize existing research findings for players, practitioners and relevant stakeholders.

### Statistical Analysis

Statistical analyses were performed using SPSS for Windows (version 29, SPSS Inc, Chicago, IL) with statistical significance accepted at *α* level of *p* < 0.05. Frequency-based metrics were reported as a percentage of the total studies. Histogram inspection revealed skewed distributed data for observational/experimental studies-specific sample sizes. As such, a Mann–Whitney *U* test was used to compare median numbers of sample sizes in observational and experimental studies, and a Kruskal–Wallis test with a post hoc Dunn test was used to assess differences in IF and Altmetric scores across observational studies, experimental studies and narrative reviews. These data were reported as median ± interquartile range (IQR).

## Results

**Fig. 2:**
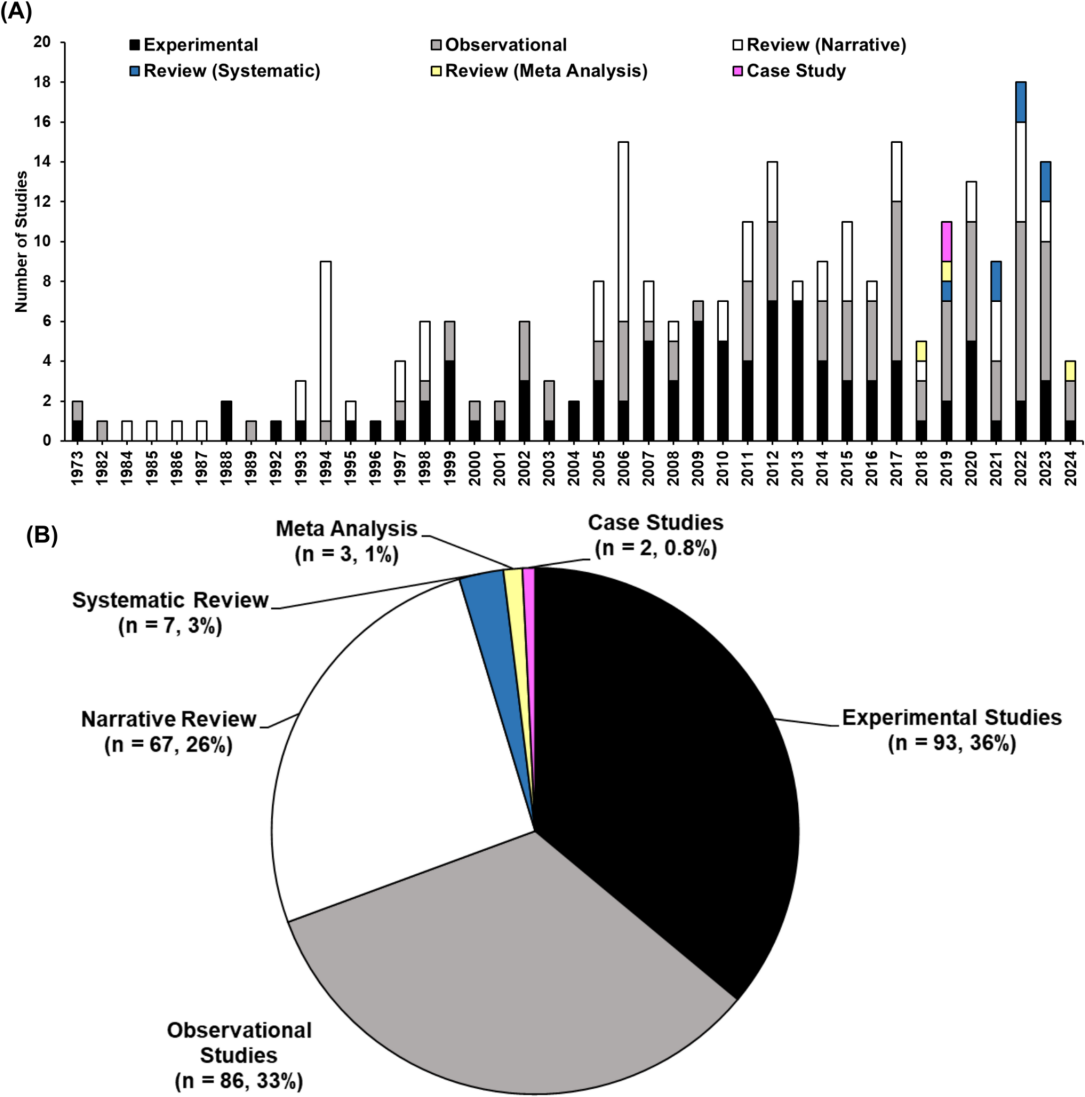
Histogram of yearly publication rate of all included studies (**A**) and the proportion of the types of studies included (**B**)

A PRISMA-ScR flow diagram was produced to report the results from the search and study selection process (Fig. 1). Of the 960 papers identified during the initial search, 229 were included. In addition, 5 papers were subsequently excluded owing to unavailable full text, resulting in a final total of 224 papers included in the review following the initial screening. From the search of the reference list, an additional 34 papers were subsequently added and, hence, a total of 258 papers were included (Fig. 1). During the audit period spanning 1973–2000, the average publication rates per year for observational studies, experimental studies and narrative reviews were 0.3, 0.6 and 0.7 studies, respectively (Fig. 2A). Subsequently, between 2001 and 2010, these rates escalated to 3.1, 1.7 and 1.7 studies per year. This trend continued with even higher rates from 2011 to 2024, reaching 3.7, 4.7 and 2.3 studies annually for observational studies, experimental studies and narrative reviews, respectively. Notably, systematic reviews, meta-analyses and case studies only emerged in publications between 2018 and 2024.

### Study Types and Research Settings

Across all papers, experimental studies (*n* = 93, 36%) were the most frequently examined study types, followed by observational studies (*n* = 86, 33%) and narrative reviews (*n* = 67, 26%), (Fig. 2B). Case studies (*n* = 2, 0.8%), systematic reviews (*n* = 7, 3%) and meta-analyses (*n* = 3, 1%) constituted the remainder of the studies. Notably, the majority of experimental studies were conducted in laboratory settings (75%), while most observational studies were conducted in field settings (98%).

### Population and Sample Size

**Fig. 3:**
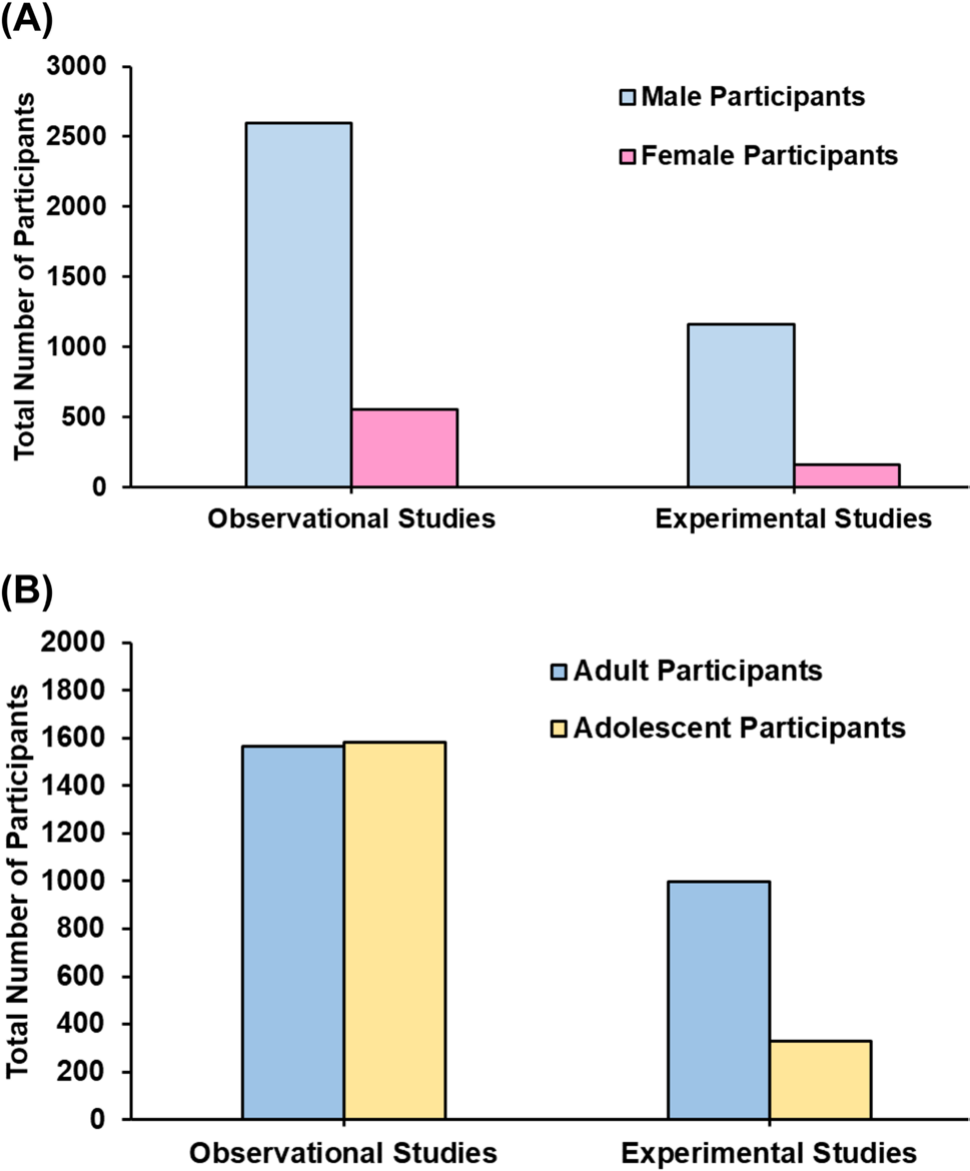
The total number of male and female participants (**A**) and the total number of adult and adolescent participants (**B**) in experimental and observational studies

There was a total of 4475 participants, with 16% being female and 84% being male. Out of the observational, experimental and case studies analysed, 78%, or 141 studies, involved male-only participants, while 12% (*n* = 22) focused solely on female participants, and the remaining 10% (*n* = 18) utilized mixed-sex cohorts. The majority of participants in observational studies (*n* = 2597, 83%), experimental studies (*n* = 1162, 88%) and case studies (*n* = 2, 100%) were male, with female participants accounting for only 18% (*n* = 552) and 12% (*n* = 162) in observational and experimental studies, respectively (Fig. 3A). Within observational studies, adults constituted 49.8% of participants (*n* = 1567), while adolescents comprised 50.2% (*n* = 1582). Conversely, in experimental studies, adults formed most participants (*n* = 996, 75%), with only 25% being adolescents (Fig. 3B). All participants in case studies were adults. In addition, the median sample size per study within experimental studies (11 ± 8) was significantly less than in observational studies (24 ± 32) (*p* < 0.001).

### Athletic Caliber and Research Topics

**Fig. 4:**
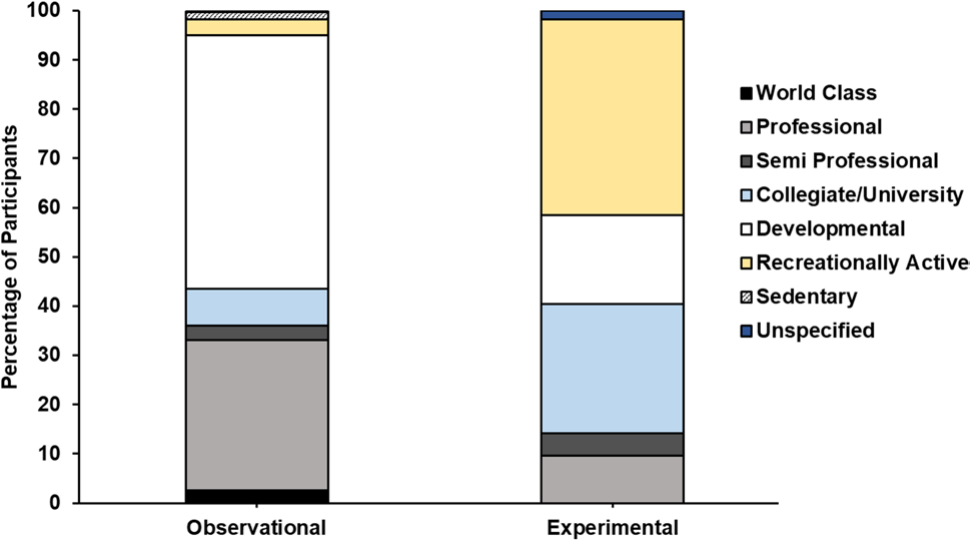
The proportion of participants in each athletic tier within observational and experimental studies

In the array of studies examined, the majority of participants were categorized as developmental (*n* = 1860, 42%) and professional (*n* = 1087, 24%) players, followed by recreationally active (*n* = 630, 14%), collegiate/university (*n* = 586, 13%), semi-professional (*n* = 152, 3%), world-class (*n* = 84, 2%) and sedentary participants (*n* = 43, 1%). The remaining participants were unspecified (*n* = 33, 0.7%). Figure 4 shows the athletic caliber of the participants in observational and experimental studies. A higher proportion of developmental and professional participants was found in observational studies in comparison with experimental studies (52% versus 25%, 31% versus 10%, respectively). Conversely, experimental studies encompassed a greater representation of recreational active (40% versus 3%) and collegiate/university participants (26% versus 8%) when contrasted with observational studies. Similar proportions of semi-professional participants were evident in observational (3%) and experimental (5%) studies. Notably, only observational studies included world-class participants (*n* = 82, 3%).

**Fig. 5:**
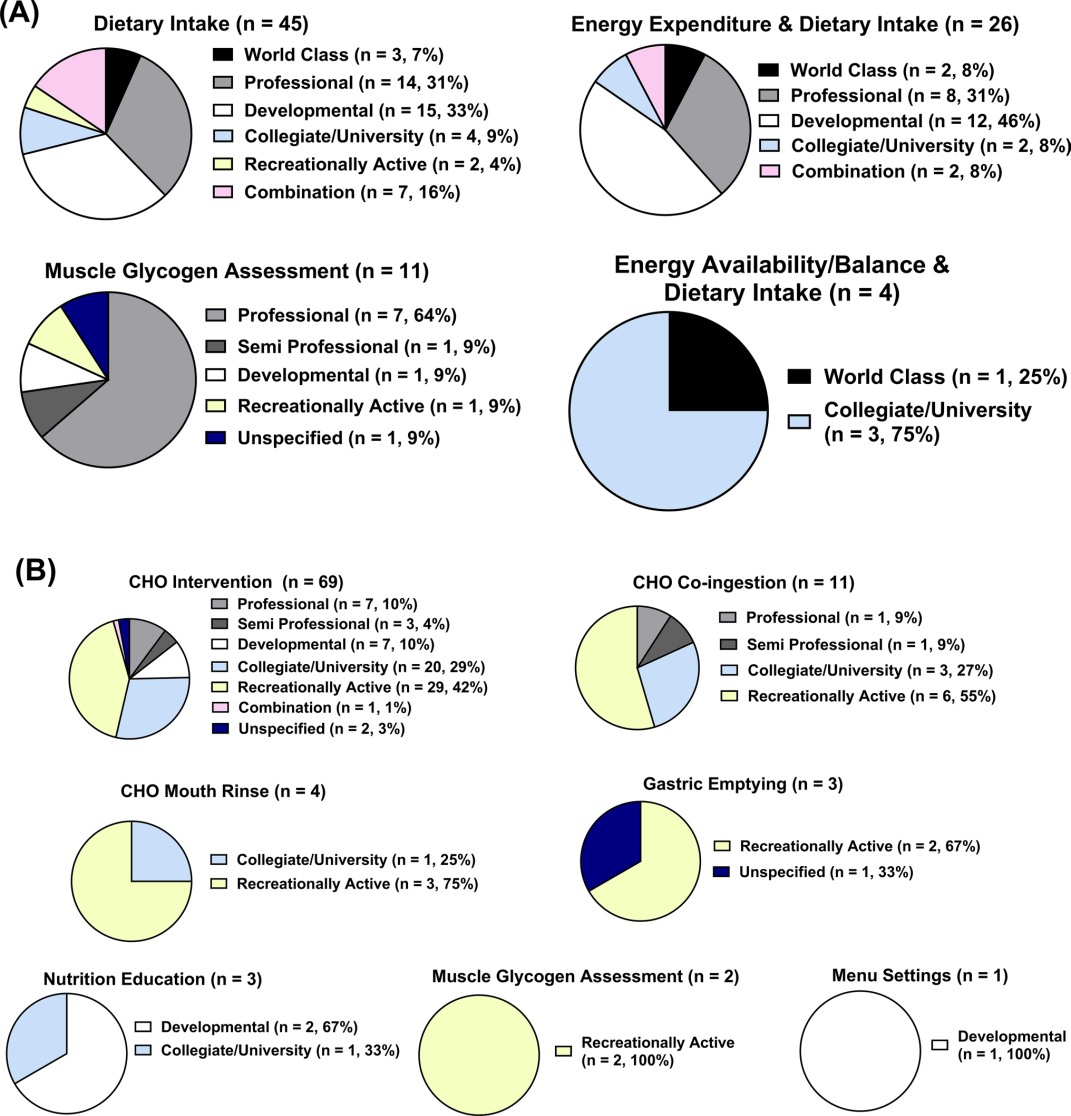
Proportion of studies within each athletic caliber for each research topic in observational studies (**A**) and experimental studies (**B**)

In observational studies, the most investigated research topic was ‘dietary intake’ (*n* = 45, 52%), followed by ‘energy expenditure and dietary intake’ (*n* = 26, 30%), ‘muscle glycogen assessment’ (*n* = 11, 13%) and ‘energy availability/balance and dietary intake’ at (*n* = 4, 5%). The majority of studies on the topics of ‘dietary intake’ (*n* = 15, 33%) and ‘energy expenditure and dietary intake’ (*n* = 12, 46%) were conducted in developmental players, whereas studies on ‘muscle glycogen assessment’ and ‘energy availability/balance and dietary intake’ focused mainly on professional players (*n* = 7, 64%) and collegiate/university players (*n* = 3, 75%), respectively (Fig. 5A). Furthermore, most studies focusing on ‘dietary intake’ (*n* = 22), ‘energy expenditure and dietary intake’ (*n* = 17) and ‘energy availability/balance and dietary intake’ (*n* = 2) were conducted during the in-season phase, while the majority of studies on ‘muscle glycogen assessment’ were carried out post exercise/match (*n* = 3) and pre, during and post exercise/match (*n* = 3) (Table 2).

**Table 2:** Frequency of research topics within all types of research

Research types	Research topics
Observational studies	(i) Dietary intake (*n* = 45) In-season (*n* = 22) [8, 48–68], unspecified (*n* = 7) [69–75], pre-season (*n* = 5) [76–80], during training (*n* = 2) [81, 82], seasonal changes (*n* = 2) [83, 84], concurrent training (*n* = 1) [85], international camp (*n* = 1) [86], longitudinal (*n* = 1) [87], relationship with muscle fatigue (*n* = 1) [88], Ramadan (*n* = 1) [89], relationship with nutrition knowledge (*n* = 1) [90], validation (*n* = 1) [91] (ii) Energy expenditure and dietary intake (*n* = 26) In-season (*n* = 17) [92–109], pre-season (*n* = 5) [110–114], unspecified (*n* = 2) [115, 116], international camp (*n* = 1) [117] (iii) Muscle glycogen assessment (*n* = 11) Post exercise/game (*n* = 3) [118–120], pre, during and post exercise/game (*n* = 3) [1, 121, 122], during exercise/game (*n* = 2) [123, 124], pre and post exercise/game (*n* = 1) [125], at rest (*n* = 1) [126], small sided game (*n* = 1) [127] (iv) Energy availability/balance and dietary intake (*n* = 4) In-season (*n* = 2) [128, 129], pre-season (*n* = 1) [130], seasonal changes (*n* = 1) [131]
Experimental studies	(i) Carbohydrate intervention (*n* = 69) During exercise/game (*n* = 40) [34, 132–169], pre-exercise/game (*n* = 9) [31, 170–177], carbohydrate loading (*n* = 8) [32, 33, 36, 37, 178–181], pre & during exercise/game (*n* = 6) [182–187], post exercise/game (*n* = 4) [38, 188–190], pre, during & post exercise/game (*n* = 1) [191], training adaptations (*n* = 1) [192] (ii) Carbohydrate co-ingestion intervention (*n* = 11) Protein (*n* = 8) [35, 193–199], caffeine (*n* = 2) [200, 201], chromium (*n* = 1) [202] (iii) Carbohydrate mouth rinse (*n* = 4) [203–206] (iv) Gastric emptying (*n* = 3) [207–209] (v) Nutrition education (*n* = 3) [210–212] (vi) Muscle glycogen assessment (*n* = 2) [213, 214] (vii) Menu settings (*n* = 1) [215]
Narrative review	(i) Nutritional recommendations (*n* = 33) Overview (*n* = 8) [2, 4, 216–221], practical application (*n* = 5) [222–226], female players (*n* = 4) [227–230], carbohydrate & fluid (*n* = 4) [231–234], alcohol (*n* = 1)[235], altitude, travel, cold and hot (*n* = 1) [236], carbohydrate & fat (*n* = 1) [237], cognitive performance (*n* = 1) [238], energy & carbohydrate (*n* = 1) ^[3]^, extreme environment (*n* = 1) [239], female & youth players (*n* = 1) [240], youth players (*n* = 1) [241], immune health (*n* = 1) [242], training adaptations (*n* = 1) [243] (ii) Metabolism/physiology (*n* = 13) [244–256] (iii) Carbohydrate (*n* = 11) Ergogenic effects (*n* = 7) [257–263], requirements & guidelines (*n* = 2) [264, 265], periodization (*n* = 1) [266], sports drink (*n* = 1) [267] (iv) Muscle glycogen (*n* = 4) [268–271] (v) Recovery (*n* = 3) [272–274] (vi) Dietary intake (*n* = 2) [275, 276] (vii) Half time strategies (*n* = 1) [277]
Review (systematic)	(i) Carbohydrate intervention (*n* = 3) [278–280] (ii) Dietary intake (*n* = 3) [41, 42, 281] (iii) Metabolism/physiology (*n* = 1) [282]
Review (meta- analysis)	(i) Carbohydrate intervention (*n* = 1) [283] (ii) Dietary intake (*n* = 1) [284] (iii) Muscle glycogen (*n* = 1) [285]
Case studies	(i) Energy expenditure & dietary intake (*n* = 1) [286] (ii) Nutrition during rehabilitation (*n* = 1) [287]

In experimental studies, the most frequently examined topic was ‘CHO intervention’ (*n* = 69, 74%), followed by ‘CHO co-ingestion intervention’ (*n* = 11, 12%), ‘CHO mouth rinse’ (*n* = 4, 4%), ‘gastric emptying’ and ‘nutrition education’ (both *n* = 3, 3%), ‘muscle glycogen assessment’ (*n* = 2, 2%) and ‘menu settings’ (*n* = 1, 1%). Most studies on these topics involved recreationally active participants, including ‘CHO intervention’ (*n* = 29, 42%), ‘CHO co-ingestion’ (*n* = 6, 55%), ‘CHO mouth rinse’ (*n* = 3, 75%), ‘gastric emptying’ (*n* = 2, 67%) and ‘muscle glycogen assessment’ (*n* = 2, 100%). In contrast, studies on ‘nutrition education’ (*n* = 2, 67%) and ‘menu settings’ (*n* = 1, 100%) were conducted with developmental players (Fig. 5B). Moreover, 58% of CHO intervention studies were carried out during exercise/match (*n* = 40), while ‘CHO co-ingestion intervention’ was mainly conducted in combination with protein (*n* = 8) (Table 2).

**Fig. 6:**
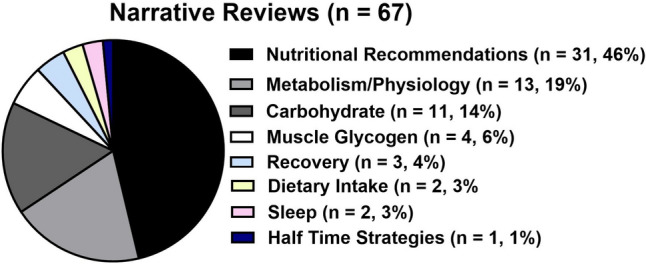
The frequency and percentage of each research topic in narrative reviews

In the narrative reviews, 46% of the papers were dedicated to presenting nutrition recommendations across various subjects (Fig. 6). In addition, 19% of the studies delved into metabolism/physiology, while 16% focused exclusively on CHO reviews. Other research topics covered in narrative reviews included ‘muscle glycogen’ (*n* = 4, 6%), ‘recovery’ (*n* = 3, 5%), ‘dietary intake’ (*n* = 2, 3%), ‘sleep’ (*n* = 2, 3%) and ‘half-time strategies’ (*n* = 1, 2%). During the audit period, systematic reviews were conducted on seven occasions, covering the topics of dietary intake (*n* = 3), CHO supplementation (*n* = 3) and metabolism/physiology (*n* = 1). In addition, meta-analyses were undertaken three times, focusing on muscle glycogen utilization, dietary intake and the impacts of CHO supplementation on mental fatigue. Lastly, case studies delved into the topics of ‘energy expenditure and dietary intake’ and ‘nutrition during rehabilitation’ (Table 2).

### Journal and Study Impact

Across all studies, the median IF of journals was 3.4 ± 2.5. Narrative reviews were published in journals with higher IF (4.1 ± 6.4) than were observational studies (3.2 ± 1.6, *p* < 0.001) and experimental studies (3.4 ± 1.6, *p* < 0.001). Altmetric scores were available for 69% of studies and were highly variable, with a median ± IQR of 12 ± 30, with no significant difference between observational studies, experimental studies and narrative reviews (*p* = 0.719). However, narrative reviews had the most studies with Altmetric scores ≥ 20 (*n* = 26), followed by experimental studies (*n* = 16) and observational studies (*n* = 14).

## Discussion

Utilizing the scoping review framework [12] and a previously published research audit tool [13], we conducted an audit of literature (both reviews and original research) with relevance to soccer-specific guidelines for CHO intake. We report that experimental studies were the most common research type (~ 36%), followed by observational studies (~ 33%) and narrative reviews (~ 26%), with experimental studies primarily conducted in laboratories, while observational studies were mostly field based. Professional and world-class players comprised ~ 33% of participants in observational studies, whereas experimental studies featured a smaller proportion, with professional players comprising only ~ 10% of participants. Moreover, no experimental studies included world-class players. Only eight experimental studies exclusively involved professional players, focusing on CHO intervention (*n* = 7) and CHO co-ingestion (*n* = 1). No field-based experimental studies have been conducted on elite players to assess the CHO requirements during matches, within immediate recovery period post matches or during different phases of the season, such as pre-season, one-game weeks and congested fixture periods. This scarcity of experimental trials among elite players contrasts with the numerous narrative reviews aimed at providing nutrition recommendations for elite players (Table 2). Our audit underscores the evidence gap in the current CHO literature for soccer, highlighting the need for more field-based experimental trials involving professional and world-class players.

Overall, ~ 30% of the studies were categorized as reviews, including narrative reviews, systematic reviews and meta-analyses, exceeding the ~ 20% previously reported in a recent audit of sports nutrition literature [5]. The prevalence of reviews in CHO literature within soccer highlights the ongoing need for original research to drive the field forward. Presently, observational and experimental studies account for ~ 33% and ~ 36% of the included studies, respectively. While carefully conducted observational studies with minimal measurement errors and innovative technologies can provide reliable and reproducible evidence on nutrition, well-designed experimental trials are essential for advancing the field [15]. Furthermore, observational studies lack the ability to establish clear causal sequence because they do not enable researchers to control for confounding variables [16]. Experimental studies, however, offer a clear comparison between intervention and control [16]. Nevertheless, only a small proportion of experimental studies were conducted in field-based settings, emphasizing the importance for practitioners and researchers to conduct more of these studies owing to its high translational potential to real-life practice [17]. Moreover, only 2 out of the 258 included studies were case studies. The underrepresentation of case studies in the CHO literature for soccer is surprising, as a rigorously conducted case study could not only offer significant translational potential into practice [17] but also provide insights into high-caliber athletes with more complex designs [18]. In addition, case studies serve as a powerful tool to bridge the gap between research and practice, facilitating communication between coaches and nutritionists. Narrative formats are easier to process, comprehend and recall and are more engaging and persuasive [18], making case studies particularly valuable in this context. Moreover, careful consideration of study quality is crucial during the research design phase, as studies with a lower risk of bias offer a more accurate representation of the true effects of an intervention [19]. To ensure this, future research should adopt rigorous methodologies, incorporate appropriate blinding techniques, use adequately sized samples, and implement strategies to minimize confounding variables, thereby reducing bias and enhancing the reliability of findings.

Females accounted for ~ 16% of the total participants in CHO literature related to soccer, mirroring the representation in recent audits of studies assessing chronic strategies to manipulate CHO intake around training [20], exercise thermoregulation [21] and heat adaptations [22]. This percentage is slightly higher compared with audits of studies examining acute CHO fueling strategies [23], but it is lower than the reported 22–71% representation of females in other audits within the field of sports science and sports medicine (SSSM) [24–27]. There is a shortage of studies on females (~ 12%), whereas studies exclusively involving male participants make up a significant percentage (~ 78%), consistent with audits of acute and chronic CHO strategies [20, 23]. Furthermore, this underrepresentation of female participants persisted across observational, experimental and case studies. Despite the limited research on female soccer players, the existing UEFA nutrition guidelines for female players remain the same as those for male players [4]. Therefore, further research on female soccer players is required to better inform and tailor these nutrition guidelines. The distribution of adults and adolescents was evenly spread across observational studies, while most participants in experimental studies were adults (~ 75%). The reduced numbers of adolescents in experimental studies could be linked with the increased emphasis on ethical concerns in research with adolescents, including issues such as obtaining assent, parental consent, risk perception and potential impact of participation [28]. Furthermore, in our audit, observational studies tended to report a greater median sample size compared with experimental studies. This necessity for large sample sizes in observational studies stems from the more heterogeneous samples, resulting in increased variability across groups [29]. Conversely, in experimental research, excessively large samples can pose challenges by significantly increasing statistical power, potentially leading to an increased likelihood of erroneously rejecting the null hypothesis [30]. Consequently, what might be considered insignificant could be falsely deemed significant. Researchers should therefore balance the needs for sufficient sample sizes to ensure statistical validity without excessively inflating the sample, which could lead to misleading results.

The majority of participants (~ 69%) in the audited studies were categorized as developmental, collegiate/university, recreationally active or sedentary. These findings are consistent with previous audits, which also indicated that most participants were classified within Tier 0 (sedentary), Tier 1 (recreationally active) and Tier 2 (developmental/trained) categories [20, 22, 23, 26, 27]. Professional and world class players made up ~ 24% and ~ 2%, respectively, of the total participants, which is higher than a previous audit in SSSM that reported ~ 9.5% from Tier 4 (elite/international) and ~ 0.5% from Tier 5 (world-class) [26]. However, only ~ 10% of participants in experimental studies were professional players, resulting in only eight studies conducted thus far with this elite cohort [31–38]. In addition, no experimental studies have been conducted on world-class players, mirroring findings from the previous audits [23, 26]. Therefore, the limited experimental research conducted on elite soccer players raises concerns about the relevance and applicability of the current nutrition guidelines to this group. Experimental studies focusing on professional soccer players have explored various timings of CHO intervention, including CHO loading [32, 33, 36, 37], pre-match [31], during intermittent exercise protocols [34], between successive 120-min matches [38] and CHO protein co-ingestion 48 h post-match [35]. Five out of these eight studies were field based [31, 33, 35, 37, 38]. Currently, no field-based experimental studies have been conducted to evaluate the effects of CHO ingestion during matches and within the immediate recovery period post-match (0–4 h). These findings align with the UEFA consensus statement, which highlights the need for more robust, sport-specific evidence tailored to the elite soccer environment. A review suggests that elite athletes may respond differently to CHO interventions compared with non-elite counterparts, as evidenced by findings that periodized CHO restriction did not enhance performance in elite endurance athletes [39]. Therefore, there is a pressing need for more randomized controlled trials on elite players to evaluate the ergogenic effects of CHO ingestion, particularly during critical time periods such as the day before the match (MD − 1), pre-match, during match and post-match and the day after the match (MD + 1). Moreover, further research is required to determine the CHO requirements of elite players during different phases of the season, including pre-season, single-game weeks and congested fixture periods.

Observational studies featured a higher representation of professional and world-class players (~ 33%) compared with experimental studies (~ 10%). A substantial portion of observational studies (~ 87%) focused on assessing dietary intake, energy expenditure or energy availability. Synthesizing these findings systematically reveals a concerning trend; both male and female soccer players, especially senior players, are failing to meet recommended CHO intake [7, 40–42]. The prevalent failure to adhere to these recommendations among players suggests a glaring disparity between research and practice. Rather than producing yet another narrative review centred solely on nutritional recommendations, which constituted ~ 46% of the narrative reviews analyzed, it becomes imperative to shift our focus towards understanding the underlying reasons for soccer players’ noncompliance with CHO intake guidelines. In this context, the Capability-Opportunity-Motivation and Behavior (COM-B) model [43] emerges as a promising framework for dissecting the multifaceted dynamics of dietary behaviours among soccer players. Recent studies employing in the COM-B model to explore the dietary behaviours of academy and female soccer players [9, 10, 44], highlight its efficacy in illuminating the complex interplay of factors influencing players’ dietary choices and habits. Thus, integrating such comprehensive models into research not only enhances our understanding but also paves the way for targeted interventions aimed at bridging the gap between CHO research in soccer and practical implementation in professional soccer players.

Our audit demonstrated that the median IF of journals in which studies were published was similar to previous audits in SSSM [22, 26, 27]. Interestingly, narrative reviews tended to be published in journals with higher IF compared with observational and experimental studies. This observation raises concerns about the incentives for researchers to pursue original research as they may receive less attention in higher-IF journals. Furthermore, our findings suggest that narrative reviews tend to garner more online attention, as indicated by a greater number of studies with Altmetric score ≥ 20 compared with observational and experimental studies. This trend is worrying, as articles with significant Altmetric attention often lead to an increased number of citations [45]. Indeed, weak positive correlation between Altmetric attention scores and citations was observed in health sciences research [46]. As a result, the lure of publishing in high IF journals and attaining heightened Altmetric attention may precipitate a surge in review articles production, mirroring recent trends in sports nutrition research over the past 5 years [5]. Although reviews offer critical synthesis, they pose the inherent risk of subjective interpretation and the potential dilution of original content through repetitive paraphrasing [6]. In light of this, to propel the field forward, researchers are encouraged to persist in their pursuit of publishing original research, notwithstanding the comparatively lower Altmetric attention and IF associated with journals more likely to publish their research.

## Conclusions

Our scoping review and audit of CHO research in soccer revealed a varied mix of study types, with experimental studies dominant in laboratory settings and observational studies prevalent in field settings. Notably, narrative reviews occupied a significant space in literature, underscoring the ongoing need for original research to advance the field. While both observational and experimental studies offer unique insights, there is a call for more field-based experimental trials in elite populations to bridge the research–practice gap. The underrepresentation of female participants in this audit highlights the need for more CHO research conducted exclusively on female soccer players. In addition, the predominance of studies focusing on nonprofessional players raises questions about the applicability of current CHO guidelines to elite soccer players. It is recommended that professional soccer clubs collaborate with research institutions over an extended period to fully integrate applied research practices, thereby addressing this gap and enhancing evidence-based practices. Research topics primarily revolved around the ergogenic effects of CHO intervention in experimental studies and dietary practices of soccer players in observational studies. However, the poor adherence of soccer players to current CHO recommendations indicates a disconnect between research and practice. Furthermore, the potential bias towards narrative reviews in journals raises concerns about the incentives for researchers. While narrative reviews may attract higher IF journals and Altmetric attention, there is a need to balance this with the pursuit of original research to drive meaningful advancements in the field. Overall, our findings highlight a lack of experimental research specific to professional and world-class soccer players for current CHO guidelines. More field-based experimental trials involving elite soccer players are necessary to provide evidence-based CHO recommendations.

## Declarations

### Availability of data and material

All data and material in this manuscript are from peer-reviewed publications.
